# Involvement of peripheral ionotropic glutamate receptors in orofacial thermal hyperalgesia in rats

**DOI:** 10.1186/1744-8069-7-75

**Published:** 2011-09-28

**Authors:** Kuniya Honda, Noboru Noma, Masamichi Shinoda, Makiko Miyamoto, Ayano Katagiri, Daiju Kita, Ming-Gang Liu, Barry J Sessle, Masafumi Yasuda, Koichi Iwata

**Affiliations:** 1 Department of Physiology, Nihon University School of Dentistry, 1-8-13 Kandasurugadai, Chiyoda-ku, Tokyo 101-8310, Japan; 2 Department of Oral and Maxillofacial Surgery, Nihon University School of Dentistry, 1-8-13 Kandasurugadai, Chiyoda-ku, Tokyo 101-8310, Japan; 3 Department of Oral Diagnosis, Nihon University School of Dentistry, 1-8-13 Kandasurugadai, Chiyoda-ku, Tokyo 101-8310, Japan; 4 Division of Oral Health Science, Dental Research Center, Nihon University School of Dentistry, 1-8-13 Kandasurugadai, Chiyoda-ku, Tokyo 101-8310, Japan; 5 Department of Anesthesiology, Nihon University School of Dentistry, 1-8-13 Kandasurugadai, Chiyoda-ku, Tokyo 101-8310, Japan; 6 Department of Psychosomatic Dentistry, Tokyo Medical and Dental University Graduate School, 1-5-45 Yushima, Bunkyo-ku, Tokyo 113-8510, Japan; 7 Department of Oral Physiology, Faculty of Dentistry, University of Toronto, 124 Edward Street, Toronto, Ontario M5G 1G6, Canada; 8 Department of Pedodontics, Nihon University School of Dentistry, 1-8-13 Kandasurugadai, Chiyoda-ku, Tokyo 101-8310, Japan; 9 Division of Functional Morphology, Dental Research Center, Nihon University School of Dentistry, 1-8-13 Kandasurugadai, Chiyoda-ku, Tokyo 101-8310, Japan; 10 Division of Applied System Neuroscience Advanced Medical Research Center, Nihon University Graduate School of Medical Science, 30-1 Ohyaguchi-Kamimachi, Itabashi-ku, Tokyo 173-8610, Japan

**Keywords:** MAP kinase, trigeminal spinal subnucleus caudalis, ionotropic glutamate receptor, sensitization

## Abstract

**Background:**

The purpose of the present study was to elucidate the mechanisms that may underlie the sensitization of trigeminal spinal subnucleus caudalis (Vc) and upper cervical spinal cord (C1-C2) neurons to heat or cold stimulation of the orofacial region following glutamate (Glu) injection.

**Results:**

Glu application to the tongue or whisker pad skin caused an enhancement of head-withdrawal reflex and extracellular signal-regulated kinase (ERK) phosphorylation in Vc-C2 neurons. Head-withdrawal reflex and ERK phosphorylation were also enhanced following cold stimulation of the tongue but not whisker pad skin in Glu-injected rats, and the head-withdrawal reflex and ERK phosphorylation were enhanced following heat stimulation of the tongue or whisker pad skin. The enhanced head-withdrawal reflex and ERK phosphorylation after heat stimulation of the tongue or whisker pad skin, and those following cold stimulation of the tongue but not whisker pad skin were suppressed following ionotropic glutamate receptor antagonists administration into the tongue or whisker pad skin. Furthermore, intrathecal administration of MEK1/2 inhibitor PD98059 caused significant suppression of enhanced head-withdrawal reflex in Glu-injected rats, heat head-withdrawal reflex in the rats with Glu injection into the tongue or whisker pad skin and cold head-withdrawal reflex in the rats with Glu injection into the tongue.

**Conclusions:**

The present findings suggest that peripheral Glu receptor mechanisms may contribute to cold hyperalgesia in the tongue but not in the facial skin, and also contribute to heat hyperalgesia in the tongue and facial skin, and that the mitogen-activated protein kinase cascade in Vc-C2 neurons may be involved in these Glu-evoked hyperalgesic effects.

## Background

It is well known from human psychophysical studies that thermal and mechanical sensitivity of the tongue is different from that of the facial skin [1-4]. Cold and heat sensory thresholds are significantly higher in the tongue compared to the facial skin. The pain threshold is also higher in tongue compared to the facial skin. Previous histological studies have also reported that cutaneous tissues are covered by orthokeratinized tissues, whereas mucosal membranes are covered by parakeratinized tissues, and mucosal surfaces are highly moisturized [5]. Furthermore, a larger number of small salivary glands are distributed in the intraoral mucosa but not in the facial skin [6]. These human psychophysical and histological data strongly suggest that thermal and mechanical sensory mechanisms are different between intraoral tissues and the facial skin.

It is also known that tissue inflammation or injury of intraoral tissues causes severe pain, such as burning pain, referred pain or chronic pain [7-9]. Following peripheral tissue inflammation or injury, a variety of inflammatory mediators, neuropeptides or adenosine triphosphate (ATP) is released from the inflamed or injured tissue [10,11]. These molecules bind specific receptors in the primary afferent neurons, resulting in sensitization of primary afferent fibers. It has also been reported that glutamate (Glu) is another candidate to activate primary afferent nociceptors following its release from inflamed or injured tissues [12-17]. The elevated concentration of Glu has also been detected in human tissues in association with chronic non-inflammatory pain conditions and may contribute to chronic deep tissue pain in humans [18,19]. It has also been reported that N-methyl-d-aspartate (NMDA) receptor antagonist ketamine injection into the temporomandibular joint (TMJ) causes significant attenuation of the Glu-induced TMJ pain in human subjects, suggesting that the ionotropic glutamate receptor is involved in Glu-induced TMJ pain [20]. These findings also suggest that Glu is released from the peripheral tissues after tissue inflammation or injury and binds to Glu receptor α-amino-3-hydroxy-5-methyl-4-isoxazolepropionic acid (AMPA) or NMDA receptor subtypes. This mechanism may be involved in the enhancement of primary afferent excitability. Some previous animal studies have revealed that the injection of Glu into the tongue [21], jaw muscles or TMJ sensitizes small-diameter primary afferent neurons innervating deep orofacial tissues and induces nociceptive processes in the central nervous system [22-25]. These findings raise the possibility that Glu may also be released peripherally after orofacial inflammation or injury and may be involved in enhancing the activity of primary afferents innervating orofacial tissues such as the tongue and facial skin. However, whether peripheral Glu receptors are involved in orofacial thermal hyperalgesia has not been investigated.

Extracellular signal-regulated kinase (ERK) is known as one of the mitogen-activated protein kinases (MAPKs) [26-28]. ERK in dorsal root ganglion (DRG) and spinal dorsal horn (DH) neurons is phosphorylated within 10 min following peripheral noxious stimuli and the number of phosphorylated ERK-immunoreactive (pERK-IR) cells increases in the DRG and DH as noxious stimulus intensity increases [29,30]. Recently, it has been reported that ERK is phosphorylated in many neurons in trigeminal spinal subnucleus caudalis (Vc) and upper cervical spinal cord (C1-C2) dorsal horn within 5 min following noxious stimulation of orofacial tissues [31,32]. These findings suggest that the activation of neurons following noxious orofacial stimulation is reflected in the phosphorylation of ERK in Vc and C1-C2 neurons, and also indicate that the ERK phosphorylation in Vc and C1-C2 neurons is a reliable marker of nociceptive neurons activated by orofacial noxious stimuli.

Thus, the aim of this study was to clarify whether peripheral Glu receptors may be involved in the central sensitization of Vc and C1-C2 neurons activated by noxious heat or cold stimulation of these orofacial tissues by using immunohistochemical technique to detect ERK phosphorylation in Vc and C1-C2 neurons following thermal stimulation of the tongue or whisker pad skin.

## Results

### Head-withdrawal reflex

**Figure 1:**
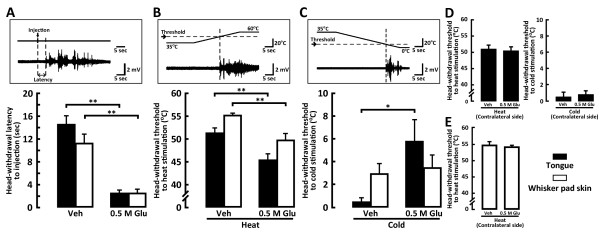
**Head-withdrawal latency to Glu or vehicle (Veh) injection into the tongue or whisker pad skin, head-withdrawal threshold to heat or cold stimulation of the tongue or whisker pad skin in the rats with Glu or Veh injection into the tongue or whisker pad skin**. The onset latency of EMG activity was recorded from the splenius capitis muscle following Glu or Veh injection into the tongue or whisker pad skin, and the head-withdrawal threshold to heat or cold stimulation of the tongue or whisker pad skin was also measured as the suprathreshold stimulus intensity evoking EMG activity. A: Mean head-withdrawal latency following Glu or Veh injection into the tongue or whisker pad skin, B and C: Mean head-withdrawal threshold following heat or cold stimulation of the tongue or whisker pad skin in the rat with Glu or Veh injection, respectively. Inset diagrams in each panel indicate EMG activities recorded from the splenius capitis muscle following Glu injection into the whisker pad skin (A), that following heat or cold stimulation of the whisker pad skin in the Glu injected rat (B and C). The head-withdrawal threshold to heat or cold stimulation of the tongue contralateral side in the rats with Glu or Veh injection into the tongue (D) and heat stimulation of the whisker pad skin contralateral side in the rats with Glu or Veh injection into the whisker pad skin (E). *: *p *< 0.05, **: *p *< 0.01.

The head-withdrawal latency following submucosal or subcutaneous Glu injection into the tongue or whisker pad skin, respectively, was significantly shorter compared to that following vehicle injection (Figure 1A). Furthermore, the head-withdrawal threshold to heat stimulation of the tongue or whisker pad skin was significantly lower in Glu-injected rats compared to that of vehicle-injected rats (tongue, Glu-injected rats: 45.47 ± 1.27°C, vehicle-injected rats: 51.28 ± 1.19°C; whisker pad skin, Glu-injected rats: 49.79 ± 1.46°C, vehicle-injected rats: 55.24 ± 1.45°C) (Figure 1B). On the other hand, the head-withdrawal threshold to cold stimulation of the tongue and whisker pad skin was different. Head-withdrawal threshold to cold stimulation of the whisker pad skin with Glu injection into the whisker pad skin was not different from that of vehicle-injected rats, whereas the head-withdrawal threshold temperature to cold stimulation of the tongue was significantly higher in Glu-injected rats compared to vehicle-injected rats (tongue, Glu-injected rats: 5.78 ± 1.92°C, vehicle-injected rats: 0.52 ± 0.34°C; whisker pad skin, Glu-injected rats: 3.49 ± 1.19°C, vehicle-injected rats: 2.95 ± 0.91°C) (Figure 1C).

The head-withdrawal threshold to heat or cold stimulation of the tongue and heat stimulation of the whisker pad skin was also measured in the contralateral side to Glu injection. We could not observe any differences in head-withdrawal thresholds to heat or cold stimulation of the contralateral tongue and heat stimulation of the contralateral whisker pad skin compared to those of vehicle injections (Figure 1D, E).

### ERK phosphorylation in Vc and C1-C2 neurons and effect of MEK 1/2 inhibitor on ERK phosphorylation

**Figure 2:**
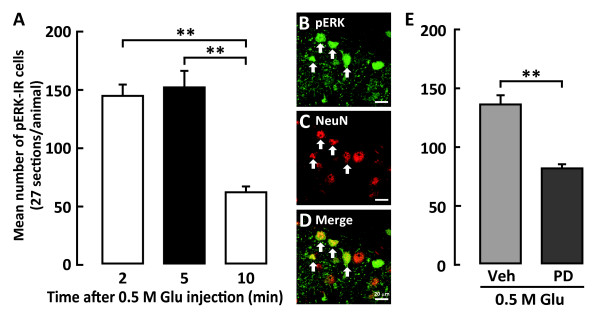
**Time-course change in the number of pERK-IR cells in the Vc and C1-C2 and the effect of i.t administration of PD98059 on ERK phosphorylation following subcutaneous Glu injection into the whisker pad skin**. A: The number of pERK-IR cells was counted at 2, 5 and 10 min after Glu injection into the whisker pad skin. B, C and D: Photomicrographs of pERK-IR cells (B), NeuN positive cells (C) and the photomicrograph of B merged with C (D). E: Mean number of pERK-IR cells in Vc and C1-C2 five min after Glu injection into the whisker pad skin in the rats with i.t. administration of PD98059 or Veh. The arrows n B, C and D indicate pERK-IR and/or NeuN positive neurons induced by Glu injection. **: *p *< 0.01.

In order to define the peak time point when the largest number of pERK-IR neurons was noted, the time course of changes in the number of pERK-IR neurons in Vc and C1-C2 was analyzed following subcutaneous injection of Glu into the whisker pad skin. ERK phosphorylation in Vc and C1-C2 neurons was detected within 2 min and peaked at 5 min following subcutaneous Glu (0.5 M) injection into the whisker pad skin (Figure 2A). Only a small number of pERK-IR cells was observed at 10 min after subcutaneous Glu (0.5 M) injection into the whisker pad skin. All pERK-IR cells also showed NeuN immunoreactivity, indicating that pERK-IR cells observed in this study were neurons (Figure 2B-D). The number of pERK-IR neurons expressed in Vc and C1-C2 after Glu injection to the whisker pad skin was significantly smaller in MEK 1/2 inhibitor PD98059-injected rats compared to that of vehicle-injected rats (Figure 2E).

**Figure 3:**
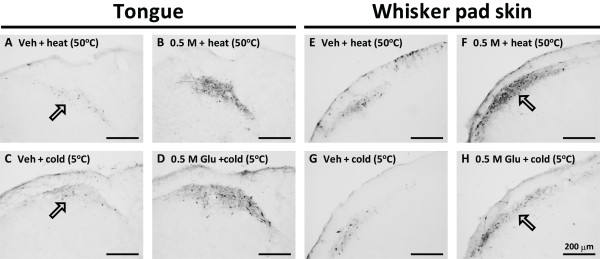
**Photomicrographs of pERK-IR cells in Vc after heat or cold stimulation of the tongue (A, B, C and D) or whisker pad skin (E, F, G and H) in the rats with Glu or Veh injection into the tongue or whisker pad skin**. Arrows in A, B, E and G indicate the areas where pERK-IR cells were observed.

A large number of pERK-IR neurons was restricted to the superficial laminae of the dorsal portion of the Vc after heat or cold stimulation of the tongue in Glu- or vehicle-injected rats (Figure 3A-D). Many were also observed in the superficial laminae of the Vc after heat or cold stimulation applied to the whisker pad skin in 0.5 M Glu- or vehicle-injected rats as indicated by the arrows (Figure 3E-H).

**Figure 4:**
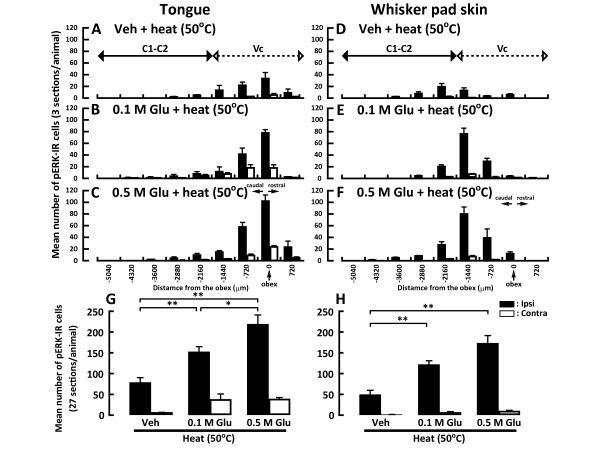
**The rostro-caudal distribution of pERK-IR cells in Vc and C1-C2 following heat stimulation of the tongue (A, B and C) or whisker pad skin (D, E and F) in the rats with Glu (0.1 M or 0.5 M) or Veh injection into the tongue or whisker pad skin**. G and H: Mean number of pERK-IR cells in Vc and C1-C2 following heat stimulation of the tongue (G) or whisker pad skin (H) in the Glu- or Veh-injected rats. Ipsi indicates ipsilateral side to Glu or Veh injection. Contra indicates contralateral side to Glu or Veh injection in this and following figures. *: *p *< 0.05, **: *p *< 0.01.

The rostro-caudal distribution of the pERK-IR neurons after heat stimulation (50°C) of the tongue or whisker pad skin with vehicle or Glu injection is illustrated in Figure 4. The number of pERK-IR neurons peaked at the obex level after heat stimulation of the tongue following submucosal injection of Glu into the tongue (Figure 4A-C). On the other hand, the number peaked at 1440 μm caudal to the obex after heat stimulation of the whisker pad skin following Glu injection into the whisker pad skin (Figure 4D-F). The number of pERK-IR neurons following noxious heat stimulation of the tongue was significantly greater in 0.5 M Glu-injected rats than in those receiving 0.1 M Glu-injected or in vehicle-injected rats (Figure 4G), and that following heat stimulation of the whisker pad skin was significantly greater in 0.5 M Glu-injected rats than that in vehicle-injected rats (Figure 4H).

**Figure 5:**
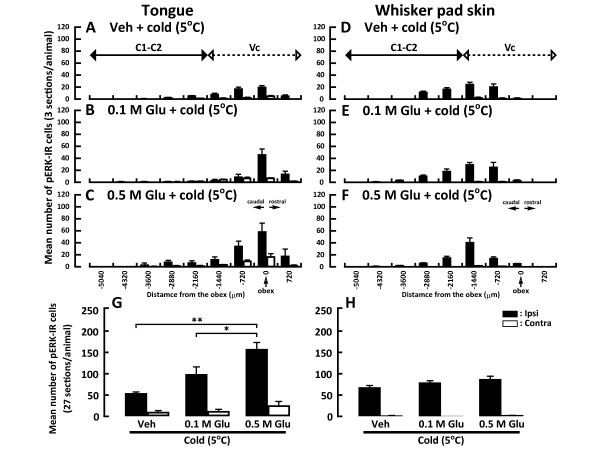
**The rostro-caudal distribution (A, B, C, D, E and F) and mean number (G and H) of pERK-IR cells in Vc and C1-C2 following cold stimulation of the tongue (A, B, C and G) or whisker pad skin (D, E, F and H) in the rats with Glu (0.1 M or 0.5 M) or vehicle injection into the tongue or whisker pad skin**. Note that significantly larger number of pERK-IR cells was observed in tongue Glu-injected rats compared with that in whisker Glu-injected rats. *: *p *< 0.05, **: *p *< 0.01.

The number of pERK-IR neurons also peaked at the obex level after cold stimulation (5°C) of the tongue following submucosal Glu (0.5 M) injection into the tongue (Figure 5A-C), whereas the number peaked at 1440 μm caudal to the obex after cold stimulation of the whisker pad skin following Glu injection into the whisker pad skin (Figure 5D-F). After cold stimulation of the tongue in vehicle- or Glu-injected rats, the number of pERK-IR neurons was significantly larger in 0.5 M Glu-injected rats following cold stimulation of the tongue compared to that of vehicle- or 0.1 M Glu-injected rats (Figure 5G). On the other hand, no differences in the number of pERK-IR neurons were observed between these three groups of vehicle- or Glu-injected rats after cold stimulation of the whisker pad skin (Figure 5H).

**Figure 6:**
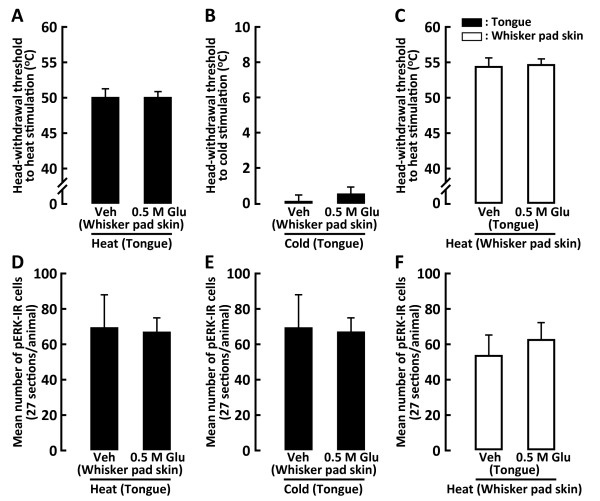
**The head-withdrawal threshold to heat or cold stimulation of the tongue in the rats with 0.5 M Glu or vehicle injection into the whisker pad skin (A, B) and heat stimulation of the whisker pad skin in the rats with 0.5 M Glu or Veh injection into the tongue (C)**. Mean number of pERK-IR cells in Vc and C1-C2 following heat or cold stimulation of the tongue in the rats with 0.5 M Glu or vehicle injection into the whisker pad skin (D, E) and heat stimulation of the whisker pad skin in the rats with 0.5 M Glu or vehicle injection into the tongue (F).

The head-withdrawal threshold and expression of pERK-IR cells in Vc and C1-C2 to heat or cold stimulation of the tongue in whisker pad Glu-injected rats and that to heat stimulation of the whisker pad skin in tongue Glu-injected rats were also analyzed, and we could not observe any significant differences in the head-withdrawal threshold and expression of the pERK-IR cells between Glu-injected and vehicle-injected rats (Figure 6A-F).

### Effect of Glu receptor antagonists on head-withdrawal reflex and ERK phosphorylation

**Figure 7:**
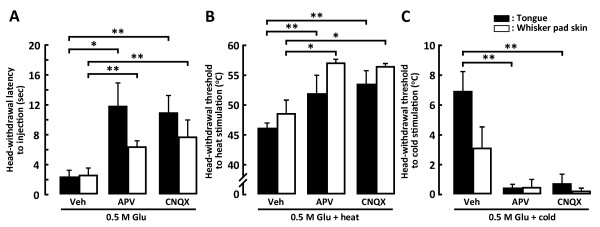
**Head-withdrawal latency to 0.5 M Glu and Veh, 1 mM APV or 10 μM CNQX injections into the tongue or whisker pad skin (A), head-withdrawal threshold to heat (B) or cold (C) stimulation of the tongue or whisker pad skin in the rats with Veh, APV or CNQX injections into the tongue or whisker pad skin**. A: Mean head-withdrawal latency following Glu or Veh injection into the tongue or whisker pad skin in the rats with Veh, APV or CNQX injection, B and C: Mean head-withdrawal threshold following heat (B) or cold (C) stimulation of the tongue or whisker pad skin in the rat with Glu and Veh, APV or CNQX injections. *: *p *< 0.05, **: *p *< 0.01.

The NMDA receptor antagonist, APV (1 mM) or non-NMDA Glu receptor antagonist, CNQX (10 μM) or vehicle was injected into the tongue or whisker pad skin. Five min later, 0.5 M Glu was injected at the same site where APV, CNQX or vehicle was injected, and the head-withdrawal latency was measured. The head-withdrawal latency to the onset of Glu injection into the tongue or whisker pad skin was significantly longer in APV- or CNQX-injected rats compared to vehicle-injected rats (Figure 7A). In Glu-injected rats, head-withdrawal threshold to heat stimulation of the tongue or whisker pad skin was also significantly higher in APV- or CNQX-injected rats compared to vehicle-injected group (tongue, APV-injected rats: 51.89 ± 1.59°C, CNQX-injected rats: 53.47 ± 1.37°C, vehicle-injected rats: 46.19 ± 1.63°C; whisker pad skin, APV-injected rats: 57.03 ± 0.68°C, CNQX-injected rats: 56.51 ± 0.43°C, vehicle-injected rats: 48.68 ± 2.13°C) (Figure 7B). The threshold temperature to evoking head-withdrawal to cold stimulation of the tongue was also significantly lower in APV- or CNQX-injected rats with Glu injection compared to those in vehicle-injected rats with Glu injection, whereas the head-withdrawal threshold to cold stimulation of the whisker pad skin was not significantly different in Glu-injected rats following APV, CNQX or vehicle injection (tongue, APV-injected rats: 0.45 ± 0.25°C, CNQX-injected rats: 0.73 ± 0.63°C, vehicle-injected rats: 6.93 ± 1.33°C; whisker pad skin, APV-injected rats: 0.51 ± 0.51°C, CNQX-injected rats: 0.23 ± 0.23°C, vehicle-injected rats: 3.13 ± 1.40°C) (Figure 7C).

**Figure 8:**
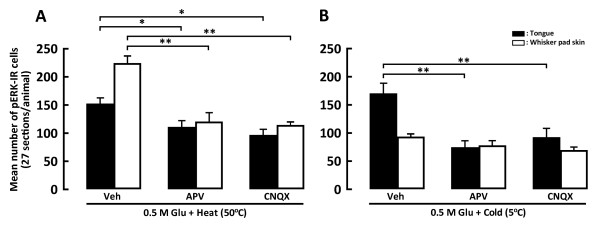
**Mean number of pERK-IR cells in Vc and C1-C2 following heat (A) or cold (B) stimulation of the tongue or whisker pad skin in the rats with 0.5 M Glu and Veh, 1 mM APV or 10 μM CNQX injections into the tongue or whisker pad skin**. Note that the mean number of pERK-IR cells following cold stimulation of the tongue but not whisker pad skin was significantly smaller in the rats with Glu- and APV- or CNQX-injected rats than that with Veh injected. *: *p *< 0.05, **: *p *< 0.01.

The number of pERK-IR neurons following heating of the tongue or whisker pad skin was significantly smaller in Vc and C1-C2 of Glu-injected rats following APV or CNQX injection compared to that following vehicle injection (Figure 8A). The number of pERK-IR neurons following cold stimulation of the tongue was significantly smaller in Glu-injected rats after APV or CNQX injection compared to those after vehicle injection (Figure 8B), but was not significantly different between APV and CNQX-injected and vehicle-injected rats following cold stimulation of the whisker pad skin (Figure 8B).

### Effect of MEK1/2 inhibitor on head-withdrawal reflex

**Figure 9:**
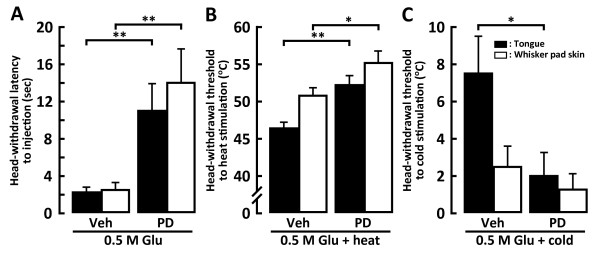
**Head-withdrawal latency to 0.5 M Glu or Veh injection into the tongue or whisker pad skin following i.t. administration of PD98059 or Veh (A), and head-withdrawal threshold to heat (B) or cold (C) stimulation of the tongue or whisker pad skin in the rats with Glu or Veh injection into the tongue or whisker pad skin following i.t. administration of PD98059 or Veh**. A: Mean head-withdrawal latency following Glu or Veh injection into the tongue or whisker pad skin in the rats with i.t. administration of PD98059 or Veh, B and C: Mean head-withdrawal threshold following heat (B) or cold (C) stimulation of the tongue or whisker pad skin in the rats with i.t. administration of PD98059 or Veh. *: *p *< 0.05, **: *p *< 0.01.

We also studied the effect of the i.t. administration of PD98059 on head-withdrawal reflex in Glu-injected rats. The head-withdrawal latency after Glu injection into the tongue or whisker pad skin was significantly longer in PD98059-injected rats compared to vehicle-injected rats (Figure 9A). The head-withdrawal threshold to heat stimulation of the tongue or whisker pad skin was significantly higher in PD98059-injected rats compared to vehicle-injected rats (tongue, PD98059-injected rats: 52.22 ± 1.28°C, vehicle-injected rats: 46.44 ± 0.83°C; whisker pad skin, PD98059-injected rats: 55.24 ± 1.46°C, vehicle-injected rats: 50.82 ± 0.98°C) (Figure 9B). The head-withdrawal threshold to cold stimulation of the tongue was also significantly lower in PD98059-injected rats compared with that of vehicle-injected rats, whereas that to cold stimulation of the whisker pad skin was not significantly different between PD98059-injected rats and vehicle-injected (tongue, PD98059-injected rats: 2.03 ± 1.22°C; vehicle-injected rats: 7.54 ± 1.95°C; whisker pad skin, PD98059-injected rats: 1.28 ± 0.81°C; vehicle-injected rats: 2.50 ± 1.11°C) (Figure 9C).

## Discussion

We have provided the first documentation of the possible involvement of peripheral Glu receptors in heat hyperalgesia of the tongue and whisker pad skin, and in cold hyperalgesia of the tongue but not whisker pad skin. Glu injection into the tongue or whisker pad skin caused significant enhancement of head-withdrawal reflex. The head-withdrawal reflex was also significantly enhanced following cold stimulation of the tongue in rats receiving Glu injection into the tongue, but was not affected by cold stimulation of the whisker pad skin in rats receiving Glu injection into the whisker pad skin. The enhancement of heat and cold head-withdrawal reflex in tongue Glu-injected rats was suppressed following peripheral administration of AMPA or NMDA receptor antagonist. Furthermore, many pERK-IR neurons were observed in Vc and C1-C2 following heat stimulation of the tongue or whisker pad skin with Glu injection, whereas a greater number of pERK-IR neurons was expressed after cold stimulation of the tongue but not whisker pad skin in Glu-injected rats. The increased number of pERK-IR cells following heat or cold stimulation of the tongue or whisker pad skin in Glu-injected rats was significantly suppressed by APV or CNQX administration. The increased number of pERK-IR cells after cold stimulation of the tongue was also significantly suppressed following APV or CNQX administration, whereas that after cold stimulation of the whisker pad skin was not altered after APV or CNQX administration. The enhanced heat head-withdrawal reflex in the tongue or whisker pad skin, and enhanced cold head-withdrawal reflex in the tongue were significantly suppressed by i.t. administration of PD98059 in Glu-injected rats. These findings suggest that peripheral Glu receptors are differentially involved in mechanisms underlying thermal hyperalgesia in tongue and whisker pad skin.

### Enhancement of head-withdrawal reflex following Glu-treatment

We documented that the head-withdrawal latency was significantly decreased following Glu injection into the tongue or whisker pad skin compared with that following vehicle injection. The enhanced head-withdrawal reflex in the rats with Glu injection was suppressed by peripheral injection of CNQX, APV or i.t. administration of PD98059. Glu injection into the human masseter muscle or TMJ is known to cause a severe pain around the injection sites as well as over a wide area of the lateral facial skin [18,20,33]. Some previous studies have also reported that Glu injection into the TMJ causes the strong enhancement of reflex responses associated with increased masseter and digastric muscle activities in rats [34,35]. It has also been reported that trigeminal afferent activity is significantly increased following Glu injection into the masseter muscle or TMJ [17,36]. They have also reported that peripheral administration of the NMDA receptor antagonist causes significant reduction of primary afferent activity, suggesting that NMDA receptor mechanism is involved in peripheral sensitization following Glu injection into the masseter muscle [37]. Our head-withdrawal reflex data is consistent with these results since head-withdrawal reflex is significantly enhanced following submucosal or subcutaneous Glu injection respectively into the tongue or whisker pad skin, supporting these recent findings that Glu activates craniofacial nociceptors. Further studies are needed to evaluate the functional differences of AMPA and NMDA receptors involved in Glu-induced peripheral thermal sensitization.

Furthermore, we could not observe any obvious extraterritorial hypersensitivity to heat or cold stimulation of the tongue in the rats with Glu injection into the whisker pad skin and heat stimulation of the whisker pad skin in Glu injection into the tongue. This suggests that peripheral Glu injection may cause allodynia or hyperalgesia in the localized area where Glu was injected.

### Tongue heat- or cold-hyperalgesia after Glu treatment

We observed that injection of APV or CNQX into the tongue or whisker pad skin significantly depressed the Glu-induced head-withdrawal reflex and enhancement of heat head-withdrawal reflex, and that head-withdrawal reflex to cold stimulation of the tongue but not whisker pad skin was significantly reduced following peripheral injection of CNQX or APV in Glu-injected rats. These findings suggest that Glu receptors are differentially involved in thermal sensitization of tongue versus facial skin.

It has been reported that transient receptor potential (TRP) vanilloid 1 (TRPV1) and TRP melastatin 8 (TRPM8) or TRP ankyrin 1 (TRPA1) are distributed in C-fiber DRG neurons and are involved in response to heat or cold stimulation of the neuronal receptive fields, respectively [38-40]. Some previous anatomical studies have also documented that TRPV1 and Glu receptors are co-expressed in C-fiber trigeminal ganglion (TG) neurons innervating the whisker pad skin or tongue [37], suggesting that these receptors co-expressed in TG neurons are involved in sensitization of heat-responsive C-fibers. It has also been documented that AMPA and NMDA receptors are expressed in DRG neurons and accumulate in C-fiber terminals [41-44]. Although our study did not determine the location of the peripheral ionotropic glutamate receptors, which have been shown to occur on afferent terminals as well as on other peripheral tissue cells [45,46], nonetheless the above-mentioned findings along with our own data suggest that peripheral Glu receptors are differentially involved in sensitization of heat and cold receptors in tongue versus whisker pad skin. Together with the previous primary afferent data, our results also suggest the following two possible mechanisms: One mechanism is that cold and heat receptors are co-expressed with Glu receptors in C-fiber TG neurons innervating the tongue, whereas TG neurons with heat and Glu receptors innervate the facial skin, resulting in the enhancement of cold and heat responses of Vc and C1-C2 neurons after Glu injection into the tongue, and the enhancement of heat response of those neurons after Glu injection into the facial skin, and the other mechanism is that these receptors are expressed in different primary afferent neurons innervating the tongue or facial skin and that converge onto Vc and C1-C2 neurons. In the case of the whisker pad skin, our data suggest that cold and Glu receptors are not co-expressed in TG neurons, and that they are expressed in different primary afferent neurons contacting on different Vc and C1-C2 neurons. Further support for the differential involvement of Glu and cold receptors on tongue versus facial skin comes from our ERK data.

### Involvement of ERK phosphorylation in tongue hyperalgesia

A large number of pERK-IR neurons was expressed in the Vc and C1-C2 after Glu injection into the tongue or whisker pad skin. The number of pERK-IR neurons following heat stimulation of the tongue or whisker pad skin was significantly greater in tongue or whisker Glu-injected rats compared with that in vehicle-injected rats. The ERK phosphorylation in Vc and C1-C2 neurons was significantly enhanced following cold stimulation of the tongue in tongue Glu-injected rats, but were not affected by cold stimulation of the whisker pad skin in whisker Glu-injected rats. The ERK phosphorylation following heat stimulation of the tongue or whisker pad skin and cold stimulation of the tongue was significantly suppressed following subcutaneous or submucosal injection of APV or CNQX. We also observed that i.t. administration of MEK1/2 inhibitor PD98059 caused significant inhibition of the head-withdrawal reflex after Glu injection itself into the tongue or whisker pad skin, and also inhibited the heat head-withdrawal reflex and ERK phosphorylation in Glu-injected rats. Cold head-withdrawal reflex after Glu injection into the tongue but not whisker pad skin was also significantly suppressed by i.t. PD98059 administration.

It is well known that ERK is phosphorylated in DRG and TG neurons after noxious stimulation of the hind paw via Ca^2+ ^influx [47], suggesting that i.t. PD98059 administration likely blocks Ca^2+ ^influx in Vc and C1-C2 neurons and TG primary afferent terminals which would contribute to the block of heat-evoked head-withdrawal reflex with Glu injection into the tongue or whisker pad skin, and to the blockade of the cold-evoked head-withdrawal reflex in tongue Glu-injected rats.

We could not observe any differences in the suppressive effect on head-withdrawal reflex to heat stimulation of the tongue and whisker pad skin and ERK phosphorylation between CNQX and APV administration in Glu-injected rats. On the other hand, the effect of CNQX and APV on cold head-withdrawal reflex in Glu-injected rats was different between the tongue and whisker pad skin. It is likely that AMPA and NMDA receptor subtypes are similarly involved in Glu-induced ERK phosphorylation in Vc and C1-C2 neurons following heating the tongue and whisker pad skin, but are differentially involve in Glu-induced ERK phosphorylation following noxious cold stimulation of the tongue and whisker pad skin, consistent with our head-withdrawal reflex findings.

## Conclusions

The present findings revealed that Glu application to the tongue or whisker pad skin caused an enhancement of head-withdrawal reflex and ERK phosphorylation in Vc and C1-C2 neurons to heat stimulation; head-withdrawal reflex and ERK phosphorylation were also enhanced following cold stimulation of the tongue but not whisker pad skin in Glu-injected rats. These findings suggest that peripheral Glu receptor mechanisms may contribute to cold hyperalgesia in the tongue but not in the facial skin, and also contribute to heat hyperalgesia in the tongue and facial skin, and that the mitogen-activated protein kinase cascade in Vc and C1-C2 neurons may be involved in these Glu-evoked hyperalgesic effects.

## Methods

### Animals

A total of 408 male Sprague-Dawley rats weighting 200-250 g were used. This study was approved by the Animal Experimentation Committee at the Nihon University. All surgeries and animal care were conducted in accordance with the National Institutes of Health Guide for the Care and Use of Laboratory Animals and the guidelines for Institutional Animal Care, and the guidelines of the International Association for the Study of Pain [48].

### Head-withdrawal reflex testing

Rats were lightly anesthetized with 2% isoflurane (Mylan, Canonsburg, PA) with 100% O_2_. As previously described [49], bipolar enamel-coated silver wire electrodes (Narishige, Tokyo, Japan) were placed in the left splenius capitis muscle for electromyographic (EMG) recordings to detect the time point of head movement (inter-electrode distance: 5-6 mm). Glu (Sigma-Aldrich, St. Louis, MI) was dissolved in isotonic saline to produce a 0.5 M Glu solution. Isotonic saline was used as the vehicle control. Five min after 0.5 M Glu (10 μl) or vehicle (10 μl) was injected with 27 gage fine needle into center of the left whisker pad skin or lateral edge of the tongue subcutaneously (depth: 2 mm), heat (1°C/sec, cut-off temperature: 60°C) or cold (1°C/sec, cut-off temperature: 0°C) stimulation was applied to the same skin or tongue site received Glu or vehicle injection (n = 6 in each group). The thermal stimuli were delivered by a contact thermal probe (5 mm in diameter, adapting temperature: 35°C, Intercross, Tokyo, Japan) in the ipsilateral or contralateral side to the Glu or vehicle injection. The head-withdrawal latency to Glu or vehicle injection into the whisker pad skin or tongue was calculated by measuring the onset latency of splenius capitis muscle EMG activity after Glu or vehicle injection. The head-withdrawal threshold to heat or cold stimulation 5 min after Glu injection was determined as the stimulus intensity when small EMG activity could be recorded after thermal stimulation as illustrated in the inset of Figure 1A. Measurement of the head-withdrawal latency to Glu injection or vehicle and head-withdrawal threshold following thermal stimulation in Glu-injected rats was conducted only once in each rat by the same investigator to ensure standardization.

The head-withdrawal threshold to heat or cold stimulation of the tongue in whisker pad Glu-injected rats and heat stimulation of the whisker pad skin in tongue Glu-injected rats were also measured (n = 5 in each group).

Although we injected the very small amount of Glu into the whisker pad or tongue, we cannot rule out the possibility of Glu entering in other body areas through the systemic circulation.

### pERK immunohistochemistry

To determine the time course and peak time of ERK phosphorylation, rats were anesthetized with sodium pentobarbital (50 mg/kg, i.p., Kyoritsu Seiyaku, Tokyo, Japan) and were perfused through the aorta with saline (500 ml) followed by 4% paraformaldehyde (Wako, Osaka, Japan) in 0.1 M phosphate buffer (PB, pH 7.4, 500 ml) at 1, 5 or 10 min after 0.5 M Glu was injected into the whisker pad skin with a fine needle (n = 5 in each time point). The Vc-C2 area was removed and post-fixed in 4% paraformaldehyde for 1 day at 4°C. The tissues were then transferred to 20% sucrose (Wako) in saline for overnight for cryoprotection. Thirty μm thick sections were cut with a freezing microtome (Leica Microsystems Japan, Tokyo, Japan). Every 4th section was collected in phosphate-buffered saline (PBS, pH 7.4). Free-floating tissue sections were dipped in 10% normal goat serum (NGS, Millipore, Billerica, MA) in PBS for 12 hours at 4°C, and then incubated in rabbit anti-Phospho-p44/42 MAP Kinase (Thr202/Tyr204) antibody (1 : 1000, Cell Signaling, Danvers, MA) for 72 hours at 4°C. Next, the sections were incubated in biotinylated goat anti-rabbit IgG (1 : 600; Vector Laboratories, Burlingame, CA) for 2 hours at room temperature. After washing in PBS, the sections were incubated in peroxidase-conjugated avidin-biotin complex (1 : 100; Vector Laboratories) for 2 hours at room temperature. They were then washed in 0.05 M tris buffer (TB, pH 7.4), and dipped in 0.035% 3,3'-diaminobenzidine-tetra HCl (Sigma-Aldrich), 0.2% nickel ammonium sulfate (Wako) and 0.05% hydrogen peroxide in 0.05 M TB. The sections were then washed in PBS, and serially mounted on gelatin-coated slides. The slides were dipped in a series from 50 to 100% alcohols after dehydrated and then cover slipped.

Next, in order to evaluate the expression of pERK-IR neurons following thermal stimulation, at 5 min after 0.1 M or 0.5 M Glu or vehicle was injected into the whisker pad skin or the tongue, heat (50°C, duration: 60 sec, interval: 10 sec, train: 5) or cold (5°C, duration: 60 sec, interval: 10 sec, train: 5) stimulation was applied to the whisker pad skin or the tongue at the site of Glu or vehicle injection through a contact thermal probe (n = 5 in each group). Five min after stimulation, rats were perfused and pERK immunohistochemistry was carried out, as described above. To study the effect of Glu injection on pERK-IR cell expression, rats were also perfused 5 min after Glu or vehicle injection into the whisker pad skin and tongue, and Vc and C1-C2 sections were processed for pERK immunohistochemistry (n = 5 in each group).

The pERK-IR neurons were counted by Neurolucida Explorer (MicroBrightField, Williston, VT). Every 720 μm sections and 2 sections before and after 720 μm were collected and the mean number of pERK-IR neurons from these 3 sections was neurons calculated, and the graphs of rostro-caudal distribution of pERK-IR neurons were prepared. The mean number of pERK-IR neurons in all the sections (27 sections/rat) was also calculated for each animal and the mean number of pERK-IR neurons in each group was compared.

Double immunofluorescence histochemistry was also used to determine if the pERK-IR neurons expressed or not a neuronal label. Five min after 0.5 M Glu injection into the whisker pad skin, heat stimuli (50°C) were applied at the site of the Glu injection (n = 5). Five min after heat stimulation, rats were perfused through the aorta with saline (500 ml) followed by 4% paraformaldehyde in 0.1 M PB (pH 7.4, 500 ml). The tissues were then transferred to 20% sucrose (Wako) in saline for overnight for cryoprotection. Thirty μm thick sections were cut and processed for double-labeling immunohistochemistry for pERK and the neuronal label NeuN. Free-floating tissue sections were dipped in 10% NGS in PBS for 12 hours at 4°C, and then incubated in rabbit anti-Phospho-p44/42 MAPK Antibody (1 : 300) for 72 hours at 4°C. Next, the sections were mouse anti-NeuN Antibody (1 : 1000, Chemicon, Temecula, CA) for 1 hour at room temperature and secondary antibodies (FITC- and rhodamine-, 1 : 100; Jackson Immuno Research, West Grove, PA) conjugated for 1 hour at room temperature in a dark room. Then the sections were washed in PBS 3 times for 5 min. The sections were mounted on slides and cover slipped in PermaFluor (Sigma-Aldrich). pERK-IR neurons and NeuN immunoreactive neurons were observed by confocal microscope (Carl Zeiss, Tokyo, Japan).

The expression of pERK-IR cells in Vc and C1-C2 to heat or cold stimulation of the tongue in whisker pad Glu-injected rats and heat stimulation of the whisker pad skin in tongue Glu-injected rats were also analyzed (n = 5 in each group).

### The effect of injection of Glu receptor antagonist APV or CNQX

Rats were anesthetized with 2% isoflurane with 100% O_2 _(for behavioral tests) or sodium pentobarbital (50 mg/kg, i.p., for immunohistochemistry). The NMDA receptor antagonist APV (Research Biochemicals International, Natick, MA), and the AMPA receptor antagonist CNQX (Research Biochemicals International) were dissolved in isotonic saline to produce a 1 mM APV and a 10 μM CNQX solution respectively, in accordance with previous studies [43,50]; isotonic saline was used as the vehicle. Five min after 10 μl APV, CNQX or vehicle was injected into ipsilateral whisker pad skin or tongue with a fine needle (27 gauge), and 5 min after that 0.5 M Glu was injected at the site of antagonist injection. At 5 min after Glu injection, behavioral tests were performed on ipsilateral side (n = 6 in each group), and another groups of animals were perfused for subsequent examination of pERK-IR in Vc and C1-C2 neurons (n = 5 in each group), as described above.

### The effect of intrathecal (i.t.) injection of MAPK Kinase (MEK) 1/2 inhibitor PD98059

In order to block the ERK phosphorylation in neurons and TG primary afferent terminals in the Vc and C1-C2, MEK1/2 inhibitor PD98059 (CalBiochem, San Diego, CA) was i.t. administrated. PD98059 was dissolved in 20% dimethyl sulfoxide (DMSO) at a concentration of 1 μg/μl (3.7 mM) as stock solution, and then further diluted to 0.1 μg/μl in 10% DMSO for i.t. injection, in accordance with previous studies [32,51]. A solution of 10% DMSO (in isotonic saline) was used as vehicle control. Rats were anesthetized with sodium pentobarbital (50 mg/kg, i.p.). The distance from C4-C5 to C1-C2 was measured in pilot studies and the length of a p10 polyethylene tube was adjusted for this length (n = 2). The polyethylene tube was inserted into the subdural space at the C4-C5 level and the tip of the tube was located near the C1-C2 level. After the experiments, vertebrae were removed and the tip of the tube was confirmed at C1-C2. An osmotic pressure pump was connected to the tube and placed subcutaneously on the back. PD98059 or vehicle was injected at a flow rate of 1.0 μl/hour for 7 days with the osmotic pressure pump. One week after a pump filled with PD98059 or vehicle pump placement, behavioral tests were performed (n = 6 in each group) and pERK-IR neurons was explored immunohistochemically (n = 5 in each group), as described above.

### Statistical analysis

Results were presented as mean ± SEM. Statistical analysis was performed using one-way analysis of variance (ANOVA) followed by Newman-Keuls test, Student's t-test or Welch's t-test as appropriate by using statistical program (SigmaStat 3.5, SYSTAT, Chicago, IL). Differences were considered significant at *p *< 0.05.

## List of abbreviations

ATP: adenosine triphosphate; Glu: glutamate; TMJ: temporomandibular joint; AMPA: α-amino-3-hydroxy-5-methyl-4-isoxazolepropionic acid; NMDA: N-methyl-d-aspartate; ERK: extracellular signal-regulated kinase; MAPKs: mitogen-activated protein kinases; DRG: dorsal root ganglion; DH: spinal dorsal horn; pERK-IR: phosphorylated ERK-immunoreactive; Vc: trigeminal spinal subnucleus caudalis; C1-C2: upper cervical spinal cord; EMG: electromyographic; PB: phosphate buffer; PBS: phosphate-buffered saline; NGS: normal goat serum; TB: tris buffer; APV: D(-)-2-amino-5-phosphonopentanoic acid; CNQX: 6-cyano-7-nitroquinoxa-line 2,3-dione; DMSO: dimethyl sulfoxide; ANOVA: analysis of variance.

## Competing interests

The authors declare that they have no competing interests.

## Authors' contributions

All authors read and approved the final manuscript. KH, NN and MS carried out the experiments and data analysis, and KH and NN were equally contributed for conducting this experiment. MM, AK, DK and ML helped the experiments, data analysis. BJS provided data interpretation and helped to finalize the manuscript. KI conceptualized the hypothesis, designed and supervised the experiments, directed the data analysis, and finalized the manuscript.
